# It's Not How Fat You Are, It's What You Do with It That Counts

**DOI:** 10.1371/journal.pbio.0060237

**Published:** 2008-09-23

**Authors:** Samuel Virtue, Antonio Vidal-Puig

## Abstract

Mechanisms underlying obesity-related metabolic disorders are poorly understood. Samuel Virtue and Antonio Vidal-Puig explore the evidence for an emerging hypothesis that attributes metabolic complications not to obesity per se, but to an individual's capacity for adipose tissue expandability.

The spiralling increase in obesity rates in the Western and developing worlds has brought with it a host of related metabolic complications including diabetes, dyslipidaemia, cardiovascular complications, and cancer. Whereas obesity itself presents its own independent health problems—such as sleep apnoea or psychological issues—the vast majority of obesity-related mortality is caused by these secondary metabolic complications. The link between obesity and such complications as insulin resistance is well established on a population level but poorly understood mechanistically. Efforts to tackle the obesity epidemic through public health initiatives and drugs have so far been notable for their lack of success. With little prospect for halting the obesity epidemic, treatment of its associated diseases becomes of paramount importance both for public health and associated costs [1].

On an epidemiological level, there is a strong correlation between obesity and diabetes; in fact, for every kilogram gained on a population level, diabetes rates increase linearly [2]. The link between obesity and diabetes has led to the assumption that the degree of insulin resistance in an individual rises in parallel with one's fat mass. However, multiple studies have demonstrated that at the individual level, the association between degree of obesity and development of insulin resistance may not be so clear cut. Several apparent clinical paradoxes appear to contradict the direct link between excessive fat mass and insulin resistance. First, it is well documented that lipodystrophic individuals develop severe insulin resistance [3,4]. Lipodystrophy is a clinical condition that is characterised by an inherent failure in adipose tissue development and/or function. Individuals with lipodystrophy cannot accumulate fat and are extremely lean, yet they often suffer from diabetes, dyslipidaemia, and other metabolic complications. Conversely, some morbidly obese individuals do not present with the Metabolic Syndrome (see Glossary) [5–7]. Furthermore, the thiazolidinedione (TZDs) class of antidiabetic drugs are potent stimulators of adipose tissue differentiation that have been demonstrated to increase body weight while also improving insulin sensitivity [8]. Resolving these paradoxes depends on understanding the mechanistic link between obesity and insulin resistance, a crucial step toward developing rational treatments for diseases such as type 2 diabetes. In this Essay, we discuss the concept of adipose tissue expandability and how it can enhance our understanding of obesity-related metabolic disease by focussing on type 2 diabetes.

**Box 1. Theories of How Obesity Causes Insulin Resistance**

**The Adipokine Hypothesis**

Obesity leads to an alteration in the profile of hormones secreted by adipose tissue (**adipokines**). In the obese state, adipose tissue secretes proportionally more adipokines that cause insulin resistance and fewer that promote insulin sensitivity.

**The Inflammation Hypothesis**

Obesity is associated with an increase in adipocyte secretion of chemokines, which promote macrophage infiltration. In addition to increased macrophage infiltration, obesity is also associated with increased macrophage activation. Activated macrophages produce cytokines that can negatively impact on insulin sensitivity.

**The Adipose Tissue Expandability Hypothesis**

Adipose tissue has a limited maximal capacity to increase in mass, which is determined on an individual basis by environmental and genetic factors. When an individual reaches their adipose tissue expansion limit, then lipid can no longer be stored appropriately in adipose tissue. Lipid that is not stored in adipose tissue is deposited in non-adipose tissue organs such as liver and muscle and causes insulin resistance by a **lipotoxic** mechanism.

The adipose tissue expandability hypothesis states that the capacity of an individual to expand their fat mass to store lipid is a more important determinant of obesity-associated metabolic problems than the absolute amount of adipose tissue an individual possesses. When an individual reaches a point where they cannot expand their adipose tissue any further, usually when they are already obese, then they present with metabolic complications due to ectopic deposition of excess lipid in non-adipose organs such as liver, muscle, and pancreatic ß cells. Ectopic deposition of lipids in organs other than adipose tissue is believed to cause insulin resistance via lipid-induced toxicity, or lipotoxicity (see Glossary).

**Glossary**

**The Metabolic Syndrome:** A collection of risk factors for cardiovascular disease that cluster together at a higher rate than can be explained by chance. The Metabolic Syndrome has multiple definitions, but all encompass components of obesity, hypertension, dyslipidaemia, and insulin resistance/diabetes. For a comprehensive review see [35].

**Lipotoxicity:** The process by which inappropriate lipid deposition in organs other than adipose tissue causes adverse affects on cellular metabolism, most notably insulin action. Specific lipids such as ceramides and diacylglyerides may be more toxic than other lipid species such as triacylglycerides.

**White Adipose Tissue:** The body's primary long-term energy store, white adipose tissue, is also responsible more acutely for whole-organism lipid homeostasis and is an important endocrine organ producing many adipokines. White adipose tissue can be subdivided into two principal forms based on anatomical location.

**Subcutaneous (SC) White Adipose Tissue** is located underneath the skin but outside the abdominal cavity. In terms of whole-organism metabolic sensitivity, SC adipose tissue is considered either beneficial or at worse inert.

**Visceral White Adipose Tissue** is located within the abdominal cavity and has been associated with a high risk of metabolic disease.

The adipose tissue expandability hypothesis suggests that the insulin resistance found in lipodystrophic and obese individuals is caused by the same pathogenic mechanism of impaired adipose tissue expansion capacity, even if the underlying cause and degree of impairment in adipose tissue expansion may be very different. While the idea that lean and very obese people may develop insulin resistance through the same pathogenic paradigm of exhaustion of adipose tissue expandability is controversial, it is actually well supported in rodent models.

## Lessons from Rodent Models

### Rodent models with impaired adipose expandability.

Several mouse studies have demonstrated that defective adipose tissue function causes severe insulin resistance [9,10]. The A/Zip fatless mouse and the adipose-nSREBP1c TG mouse both have impaired adipogenesis, which results in a lipodystrophic animal. Both mouse models demonstrate that functional adipose tissue is necessary to maintain normal carbohydrate and lipid metabolism. However, the majority of type 2 diabetic individuals are not lipodystrophic but are actually obese. Models of murine lipodystrophy do not help to answer whether limited or exhausted adipose tissue expansion can cause metabolic complications in an obese state, or whether it is exclusively a phenomenon associated with lipodystrophy.

To test if the adipose tissue expandability hypothesis can be applied to obesity-induced diabetes, it is necessary to investigate obese mouse models with subtle reductions in adipose tissue expansion. Two recent mouse studies [11,12] have demonstrated that even in overweight or obese mice, a genetic limit on adipose tissue expansion can exacerbate insulin resistance. The ob/ob mouse model, which lacks the hormone leptin, is extremely obese and insulin resistant. According to the adipose tissue expandability hypothesis, subtly limiting expansion of adipose tissue in the ob/ob mouse should increase the severity of insulin resistance while decreasing fat mass. This concept is exemplified by the PLO mouse, which carries a dominant negative mutation in the pro-adipogenic transcription factor PPARγ on an ob/ob genetic background. Interestingly, PLO mice become much more insulin resistant than ob/ob controls do, yet they have 14% less adipose tissue [11]. Despite only a subtle reduction in total fat mass compared to ob/ob controls, the severity of the insulin resistance found in the PLO mouse supports the idea that impaired adipose expansion may cause insulin resistance in the context of obesity.

### An obese but insulin-sensitive mouse model.

Based on the adipose tissue expandability hypothesis, it should be possible to become massively obese without metabolic complications, so long as new adipose tissue can be made. Until recently there was no mouse model of such a state. The recent publication by Kim et al. [13] of a mouse overexpressing adiponectin in adipose tissue on an obese ob/ob background—the AdTG-ob/ob mouse—provides the first example of a mouse with apparently limitless adipose tissue expandability. Despite having a body weight that is 50% greater than an ob/ob mouse, the AdTG-ob/ob mouse remains insulin-sensitive with no ectopic deposition of fat in liver [13]. Of note, the majority of the adipose tissue gained in the AdTG-ob/ob mouse is stored in subcutaneous depots, which in human studies have been shown to be far less detrimental to metabolic health than visceral adipose tissue stores. Thus, the AdTG-ob/ob mouse is a dramatic example of the power of increasing adipose tissue expandability to prevent metabolic complications.

## Evidence from Humans

Assaying adipose tissue expandability in humans is far more difficult than in mice; however, some indirect evidence does support the concept that once adipose tissue capacity for lipid storage is exhausted, metabolic complications ensue. Evidence for a role for adipose tissue in lipid homeostasis comes from studies of human adipose tissue function in lean and obese humans. These studies showed that adipose tissue from obese individuals was unable to appropriately take up lipids in the postprandial state and that insulin did not impair fatty acid release as efficiently as in lean individuals, suggesting that failure in lipid buffering in adipose tissue is also a result of excessive expansion of adipose tissue. The failure to buffer lipid appropriately in obese individuals leads to elevated fatty acids and triglycerides, producing a lipotoxic lipid profile that we believe is a fundamental pathogenic mechanism leading to the Metabolic Syndrome. One possible explanation for this buffering failure is that the adipose tissue has become saturated, or reached the limit of its storage capacity, in obese individuals [14].

The adipose tissue expandability hypothesis is further supported by studies that have used TZDs to treat non-alchoholic fatty liver disease (NAFL) and non-alcoholic steatohepatitis (NASH). These studies have demonstrated excellent efficacy of TZDs in terms of reduced hepatic lipid content. Interestingly, TZDs promote adipose tissue expansion and, in multiple studies, lead to weight gain. As increased body mass index (BMI) is a risk factor for NASH and NAFL, it is striking that these potent lipogenic agents actually reduce hepatic lipid accumulation [15,16]. The efficacy of TZDs appears even more contradictory when considering their molecular mechanism. In addition to their effects on body weight, TZDs might be expected to directly increase liver lipid content by activating PPAR . Many of the genes involved in both lipid uptake (such as lipoprotein lipase) and de novo fatty acid biosynthesis, such as fatty acid synthase and acetyl-CoA carboxylase, are up-regulated by PPAR . Thus the coordinate activation of lipid uptake and lipid biosynthesis by PPAR would be expected to directly increase hepatic lipid deposition (steatosis) [17,18]. However, the adipose tissue expandability hypothesis explains how TZDs can be beneficial for NASH; since increasing the capacity of adipose tissue to store fat allows repartitioning of lipid from liver back to adipose tissue and therefore ameliorates the disease state.

## Relationship to Other Hypotheses as to How Obesity Causes Diabetes

The adipose tissue expansion hypothesis should be considered one of several major hypotheses to explain the link between obesity and the Metabolic Syndrome (see Box 1). Perhaps the two most well-considered alternative hypotheses emerge from characterizing obesity as a low-grade inflammatory state and adipose tissue as an endocrine organ [19]. It has been suggested that the action of cytokines such as Tnf-a and IL6, which are increased in adipose tissue in an obese state, leads to insulin resistance. Both Tnf-a and IL6 cause insulin resistance within adipose tissue [20]. The hypothesis that obesity is a generalised low-grade inflammatory state does not necessarily contradict the adipose tissue expandability hypothesis; in fact, the adipose tissue expandability hypothesis may help to explain how obesity leads to inflammation. In humans, obesity correlates well with an increase in adipocyte size, which fits with a failure to recruit new adipocytes to expand adipose tissue depots. Skurk et al. [21] demonstrate that large adipocytes secrete proportionally more pro-inflammatory cytokines than anti-inflammatory cytokines when compared to small adipocytes, and crucially also secrete more chemoattractants associated with macrophage infiltration than smaller adipocytes. Furthermore, free fatty acids (FFAs), which are increased when adipose tissue fails to store lipid appropriately, have also been demonstrated to directly activate macrophages [22]. Thus, a failure in adipose tissue expansion may cause a “double whammy” of macrophage recruitment and activation, causing a localised and potentially systemic inflammatory state. Other pathogenic mechanisms related with insulin resistance include endoplasmic reticular (ER) stress and the effects of reactive oxygen species (ROS). Again, both ER stress and ROS are induced by FFA in cell models [23,24].

The second major hypothesis regarding how obesity leads to diabetes regards the role of adipose tissue as an endocrine organ. Adipose tissue is known to secrete multiple hormones, of which leptin is perhaps the most famous. Leptin was cloned in 1994 (and has subsequently been demonstrated to play important roles in the control of body weight and insulin sensitivity). Over the past 14 years, many more adipokines have been identified including pro-insulin sensitising adipokines such as adiponectin and adipokines that have been implicated in causing insulin resistance, such as visfatin, retinol binding protein 4, and resistin. As discussed above, adipose tissue can also secrete chemokines and cytokines. The idea that obesity leads to altered adipokine profiles that can subsequently cause systemic insulin resistance is well supported. Again, the adipokine hypothesis is not contradictory to the adipose tissue expandability hypothesis. Larger adipocytes found in the obese state have altered profiles of adipokine secretion. Furthermore, insulin resistance can cause alterations in adipokine secretion; for example, 4-month-old ob/ob mice have far less adiponectin than wild-type littermates do [11]. The data regarding adiponectin levels in ob/ob mice would suggest that impaired adipose tissue expansion may cause insulin resistance and then affect adipokine levels. Conversely adipokines themselves may modulate capacity for adipose tissue expansion. For instance, the adiponectin Ad-TG mouse model commented on above is a mouse model overexpressing an adipokine that has greatly increased adipose tissue expansion capacity [13].

## The Relative Contribution of Visceral versus Subcutaneous Adipose Tissue

So far, this Essay has discussed adipose tissue in general terms; however, in humans, there are distinct differences between adipose tissue depots dependent on their location. Different adipose tissue depots have distinct gene expression profiles [25]. Furthermore, in humans, visceral adiposity is strongly associated with increased metabolic risk, whereas subcutaneous adipose tissue is not associated with metabolic complications and may even be protective [26]. The reason for the beneficial effects of subcutaneous adipose tissue compared to visceral adipose tissue is unclear; however, it has been suggested that the proximity of visceral adipose tissue to the portal vein may allow visceral adipose tissue to exert more direct metabolic effects on the liver. Although the proximity of visceral adipose tissue to the liver suggests that it may have a disproportionate metabolic effect on this organ, even in the obese state, over 70% of FFA that are delivered to the liver are non-viscerally derived [27]. If, as we believe, a failure in FFA buffering is key component of the Metabolic Syndrome, then a failure in subcutaneous adipose tissue expandability resulting in a failure in SC lipid buffering would be expected to exert more severe metabolic effects than failure in visceral adipose tissue expansion. Importantly, a net result of maintained visceral adipose expansion with a failure in subcutaneous adipose tissue expansion would be a relative increase in visceral adiposity associated with worsened metabolic complications.

Evidence to support the concept that visceral and subcutaneous depots have different intrinsic adipose tissue expansion capacities comes from studies demonstrating that preadipocytes from subcutaneous depots differentiate more rapidly than those from omental depots and are more responsive to TZDs [28,29]. The increased adipogenic potential of the subcutaneous depot, when compared to the visceral depot, is in good accordance with the adipose tissue expandability hypothesis. In other words, metabolically better adipose tissue has greater expansion capacity than metabolically detrimental adipose tissue.

Although the different metabolic properties of visceral and subcutaneous fat may be related to the anatomical location of the fat depots, there is some evidence to suggest that there may be intrinsic cell-autonomous differences between subcutaneous and visceral adipose tissue. Perhaps the most compelling evidence for intrinsic metabolic properties of subcutaneous and visceral adipose tissue depots come from the work of Tran et al., demonstrating that transplanting subcutaneous fat into the abdominal cavity of mice improved the metabolic profile when compared to either visceral-to-visceral fat transplants, visceral-to–subcutaneous, or subcutaneous-to-visceral transplants [30].

A final aspect regarding different adipose tissue depots is why some individuals develop increased adipose tissue in abdominal depots and others in visceral depots. It is likely that the interactions between intrinsic properties of the depots [31] coupled to altered levels of hormones such as cortisol, growth hormone, sex hormones, and insulin all have a part to play [32], but more research into this aspect is necessary.

## The Concept of a Metabolic Set Point

**Figure 1:**
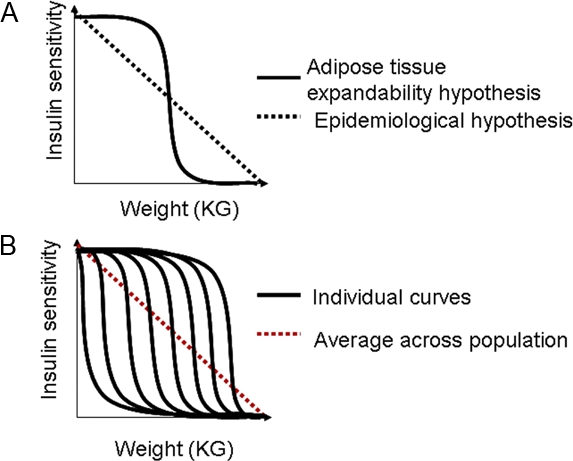
Insulin Sensitivity (A) Diagram showing the different models of how weight loss affects insulin sensitivity within an individual. (B) Diagram showing how multiple individuals with sigmoid responses in insulin sensitivity can add to form a linear alteration in insulin sensitivity across a population.

Although epidemiologically the risk of diabetes, for example, increases linearly with increased body weight, the adipose tissue expandability hypothesis suggests that this may not be the case in an individual. Rather, once an individual reaches their maximal adipose tissue mass, then metabolic complications ensue, suggesting that individuals would go from metabolically normal to metabolically compromised in a relatively small weight window (Figure 1A). When averaging effects of a large number of individuals, this could still result in a linear relationship between weight gain and reduction in insulin sensitivity (Figure 1B). Although the model presented above is an oversimplification (obesity is not normally distributed across the population), it does demonstrate how individuals can have dramatically different metabolic responses to an idealised population. While the concept of a metabolic set point is an attractive idea, it remains to be validated.

## Future Areas of Investigation

The concept that the primary mechanism linking obesity to metabolic complications is a failure in the capacity of adipose tissue to expand to accommodate excess nutrients has several clinical and scientific implications. Scientifically, it creates a unified framework to help to explain how diabetes can occur in both obese and lipodystrophic states. It also integrates explanations of how changes in lipid handling and adipocyte size may interact to lead to inflammation, dyslipidaemia, and ultimately to diabetes. However, further work remains to validate the adipose tissue expandability hypothesis and how it may relate obesity to diabetes.

The recent study of Spalding et al. [33] demonstrates that adipocyte number in the obese, though higher than in lean individuals, remains static in later life, suggesting an individualised maximum threshold for adipose tissue expansion. Furthermore alterations in adipose tissue mass in adult life involve changes in adipocyte size, rather than number. This study does not investigate whether adipocyte number and formation correlated with metabolic complications rather than just obesity per se. Crucially, during childhood and early adulthood, adipocyte number does increase dramatically; and intriguingly, this increase occurs earlier in the obese than in the lean. These processes that regulate adipocyte hyperplasia in early life need to be determined and could potentially provide new targets for the treatment of obesity-associated metabolic complications. It is important to note that this study was only concerned with people who were obese from childhood and that adipocyte number may be plastic in the case of adult-onset obesity.

The finding that adipocyte number is stabilized in adults raises many questions about how adipocyte formation and life span are regulated. If birth and death rates of adipocytes are maintained so closely, then there must be a homeostatic mechanism that allows the control of birth and death of adipocytes. But how is the formation of new adipocytes regulated? Is it at the level of commitment of mesenchymal stem cells to preadipocytes, preadipocyte hyperplasia, or differentiation of preadipocytes to mature adipocytes? Clearly a large number of questions regarding how adipose tissue plasticity is regulated remain, and understanding these processes will be essential if new drugs that target adipose tissue expandability are to be developed.

Clinically, the adipose tissue expandability hypothesis has several implications. First, it explains how pharmacological agents that promote adipose tissue expansion may be suitable for the treatment of a variety of disorders, including diabetes, NASH, NAFL, and most recently dyslipidaemia. Studies demonstrating that TZDs may be suitable for the treatment of a wide variety of metabolic disorders beyond diabetes have unfortunately coincided with concerns over the safety of this class of drugs, stressing the need for new drugs that target adipose tissue expansion.

Finally, the adipose tissue expansion hypothesis may allow for the design of better obesity treatment regimes, particularly with respect to personalised weight loss programs. If there is a “set point” for body weight and adipose tissue expansion beyond which an individual becomes metabolically compromised, then tailoring a weight loss program to an individual's threshold of metabolic complications, rather than to general therapeutically-unachievable targets based on population studies, may result in better compliance and therefore treatment efficacy [34].
